# Histone Deacetylase Inhibitors to Overcome Resistance to Targeted and Immuno Therapy in Metastatic Melanoma

**DOI:** 10.3389/fcell.2020.00486

**Published:** 2020-06-17

**Authors:** Minjeong Yeon, Youngmi Kim, Hyun Suk Jung, Dooil Jeoung

**Affiliations:** ^1^Department of Biochemistry, College of Natural Sciences, Kangwon National University, Chunchon, South Korea; ^2^Institute of New Frontier Research, College of Medicine, Hallym University, Chunchon, South Korea

**Keywords:** anti-cancer drug resistance, HDACs, immune checkpoint, MAPK, melanoma

## Abstract

Therapies that target oncogenes and immune checkpoint molecules constitute a major group of treatments for metastatic melanoma. A mutation in *BRAF* (BRAF V600E) affects various signaling pathways, including mitogen activated protein kinase (MAPK) and PI3K/AKT/mammalian target of rapamycin (mTOR) in melanoma. Target-specific agents, such as MAPK inhibitors improve progression-free survival. However, BRAF^V600E^ mutant melanomas treated with BRAF kinase inhibitors develop resistance. Immune checkpoint molecules, such as programmed death-1 (PD-1) and programmed death ligand-1(PD-L1), induce immune evasion of cancer cells. MAPK inhibitor resistance results from the increased expression of PD-L1. Immune checkpoint inhibitors, such as anti-PD-L1 or anti-PD-1, are main players in immune therapies designed to target metastatic melanoma. However, melanoma patients show low response rate and resistance to these inhibitors develops within 6–8 months of treatment. Epigenetic reprogramming, such as DNA methylaion and histone modification, regulates the expression of genes involved in cellular proliferation, immune checkpoints and the response to anti-cancer drugs. Histone deacetylases (HDACs) remove acetyl groups from histone and non-histone proteins and act as transcriptional repressors. HDACs are often dysregulated in melanomas, and regulate MAPK signaling, cancer progression, and responses to various anti-cancer drugs. HDACs have been shown to regulate the expression of PD-1/PD-L1 and genes involved in immune evasion. These reports make HDACs ideal targets for the development of anti-melanoma therapeutics. We review the mechanisms of resistance to anti-melanoma therapies, including MAPK inhibitors and immune checkpoint inhibitors. We address the effects of HDAC inhibitors on the response to MAPK inhibitors and immune checkpoint inhibitors in melanoma. In addition, we discuss current progress in anti-melanoma therapies involving a combination of HDAC inhibitors, immune checkpoint inhibitors, and MAPK inhibitors.

## Introduction

Melanoma arises from melanocytes in the skin or mucosa (Chodurek et al., 2014). Metastatic melanoma accounts for about 1–2% of skin cancers (Jiang et al., 2017). However, it is responsible for 90% of all mortality in skin cancer patients. Over the past decade, a better understanding of the molecular basis of melanoma has led to the development of anti-cancer drugs that target molecular signaling pathways that are activated in malignant metastatic melanoma.

Since 2011, several chemical inhibitors that target molecular signaling pathways have been approved by the FDA. These chemical inhibitors include vemurafenib, dabrafenib, trametinib, encorafenib, binimetinib, and cobimetinib. These anti-cancer drugs inhibit MAPK (BRAF/MEK/ERK) signaling (Pedini et al., 2019; Yarchoan et al., 2019; Ascierto et al., 2020a, b; Krayem et al., 2020). Vemurafenib improves overall survival (84% vs. 64%) and response rate (48% vs. 5%) compared with standard anti-cancer drug treatment, such as dacarbazine, in melanoma patients with the *BRAF*^*V600E*^ mutation (Chapman et al., 2011). A combination of dabrafenib and trametinib improved overall survival at 12 months compared with vemurafenib treatment (72% vs. 65%) in a phase 3 clinical trial of *BRAF*^*V600E*^ mutant melanoma patients (Robert et al., 2015). However, innate and acquired resistance to these anti-cancer drugs is a serious problem.

The tumor microenvironment plays a major role in the proliferation of melanoma cells and anti-cancer drug resistance (Guo et al., 2020). The tumor microenvironment consists of cancer cells, endothelial cells, fibroblasts, and innate and adaptive immune cells. Cancer cells interact with immune cells such as natural killer (NK) cells, macrophages (M1/M2), myeloid-derived suppressor cells (MDSCs), and cytolytic T lymphocytes (CTLs). Cancer cells can evade the antitumor response of CTLs (Freeman et al., 2019). Immune checkpoint molecules, such as PD-1 and PD-L1, regulate the interactions between cancer cells and immune cells. The interaction between PD-1 and PD-L1 leads to immune evasion of cancer cells (Hei et al., 2020). Immunotherapy aims to suppress immune evasion (tumor tolerance) by targeting the interactions between cancer cells and immune cells.

Over the last decade, immune checkpoint inhibitors (nivolumab and pembrolizumab) targeting PD-1/PD-L1 interactions have been approved by the FDA. In a clinical trial of elderly patients (>75 years old) with metastatic melanoma, nivolumab (anti-PD-1 antibody) showed clinical benefits and was well tolerated (Ridolfi et al., 2020). Pembrolizumab, an anti-PD-1 antibody, improved progression-free survival compared to BRAF inhibitors and PD-L1 inhibitors in clinical trial of stage III melanomas (Lorenzi et al., 2019). A phase Ib trial of avelumab, an anti-PD-L1 antibody, in 51 patients with stage IV unresectable melanoma showed an objective response rate (ORR) of 21.6% (Keilholz et al., 2019). Thirty-nine patients experienced side effects, including infusion-related reactions, fatigue, and chills (Keilholz et al., 2019).

Histone acetylation/deacetylation plays a critical role in the expression of genes involved in immune evasion of cancer cells (Knox et al., 2019). Histone modification is closely associated with cancer progression (Halasa et al., 2019). High expression levels of several HDACs have been associated with poor survival in cancer patients (Dembla et al., 2017). Thus, HDACs may regulate expression of PD-1 and PD-L1. These reports suggest that HDACs may be targets for the development of anti-melanoma therapies.

Herein, we review the roles of signaling pathways and immune checkpoint molecules in melanoma progression and anti-cancer drug resistance. We address the roles of HDACs in the regulation of oncogenic signaling pathways and immune evasion by cancer cells. We also discuss current progress in combination therapies that employ histone deacetylases inhibitors, targeted treatments, and immune therapy for treatment of malignant melanoma.

## The Mechanisms of Anti-Cancer Drug Resistance in Melanoma

Melanoma is a common and potentially lethal type of skin cancer. Almost half of all cutaneous melanomas have the *BRAF*^*V600E*^ gene mutation that results in activation of MAPK signaling (Feng T. et al., 2019; Rossi et al., 2019; Woo et al., 2019). *BRAF*^*V600E*^ mutant metastatic melanomas display activation of both MAPK-dependent and –independent signaling pathways for survival under MAPK inhibitor treatment in a PDX mouse model (Feng T. et al., 2019). BRAF/MEK inhibitors have some clinical benefits. However, melanoma patients develop resistance to these inhibitors within 6-8 months (Roskoski, 2018; Fujimura et al., 2019).

**FIGURE 1:**
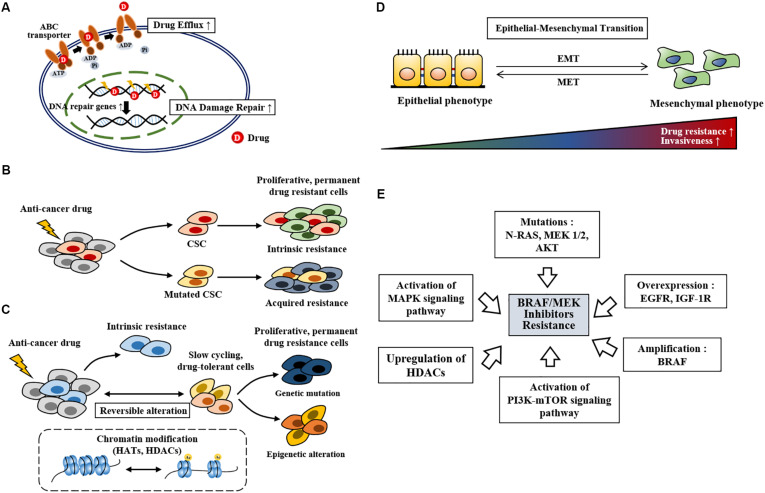
The mechanisms of anti-cancer drug resistance. **(A)** Drug efflux by ABC transporter activity, drug inactivation, and alterations in drug targets leads to anti-cancer drug resistance. Increased DNA damage repair also leads to anti-cancer drug resistance. **(B)** Cancer stem cells survive anti-cancer drug treatment. Mutations (point mutations, gene amplifications etc.) in these cancer stem cells lead to anti-cancer drug resistant phenotypes. Cancer stem cells that survive anti-cancer drug treatment proliferates and lead to anti0cancer drug resistance (intrinsic resistance). CSC denotes cancer stem cell. **(C)** Slow-cycling drug-tolerant cells are selected on treatment by reversible epigenetic reprogramming. Further epigenetic reprogramming give rise to re-proliferating drug-resistant cells. Genetic mutation in slow-cycling drug-tolerant cells also give rise to permanent drug-resistant cells. HATs denote histone acetyl transferases. **(D)** Mesenchymal transition is closely related to increased drug resistance and invasiveness. MET denotes mesenchymal-epithelial transition. **(E)** Repeated exposure to BRAF inhibitors spurs resistance. BRAF inhibitor resistance develops from gene amplification, gene overexpression, genetic mutations, activation of signaling pathways, and upregulation of HDACs.

Anti-cancer drug resistance can be classified into innate and acquired resistance. Innate resistance exists even before treatment while acquired resistance develops after treatment. Innate anti-cancer drug resistance is closely related to inherent gene mutations (Shinohara et al., 2019), drug efflux (Xiao et al., 2018, Figure 1A), and selection of cancer stem cells upon treatment (Green et al., 2019, Figure 1B). DNA damage repair (Figure 1A), phenotypic switching, epigenetic reprogramming (Figure 1C), enrichment of slow cycling cells (Figure 1C), and reactivation of molecular signaling pathways also play critical roles in anti-cancer drug resistance. High level of ABCB5 (ATP-binding cassette transporter, subfamily B, member 5) is responsible for resistance to the *BRAF* inhibitor vemurafenib (Xiao et al., 2018). Enhanced DNA damage repair by NF-κB confers resistance to chemotherapy (Li et al., 2017).

Anti-cancer drug resistance is associated with the presence of induced drug-tolerant cells (Kim et al., 2010, 2015; Al Emran et al., 2018). These induced drug-tolerant cells resulting from exposure to chemotherapy display histone lysine modifications, which are characteristic of epigenetic reprogramming (Al Emran et al., 2018). Exposure to vemurafenib enriches slow cycling melanoma cells expressing H3K4-demethylase JARID1B (Roesch et al., 2013). Inhibition of mitochondrial function enhances sensitivity to vemurafenib by decreasing the expression of JARID1B (Roesch et al., 2013). Rapidly proliferating cancer cells, but not slow cycling cells, are the main subjects of targeted therapy. Slow cycling cancer cells are enriched by anti-cancer drugs and confer resistance by activating various signaling pathways, including the WNT5A and EGFR pathways (Ahn et al., 2017). Figure 1 shows the mechanisms of anti-cancer drugs resistance. Tumor heterogeneity and plasticity (phenotypic switching) are responsible for resistance to various anti-cancer drugs (Su et al., 2019, Figure 1D). Tumor heterogeneity includes cell type heterogeneity and genetic heterogeneity. These characteristics make it almost impossible to rely on a single therapy for cancer treatment. Melanoma cells switch between differentiated (proliferative) and de-differentiated (invasive) states during metastatic progression. Phenotypic switching toward the de-differentiated state leads to resistance to *BRAF* and *MEK* inhibitors (Granados et al., 2020). BRAF inhibitor treatment induces mesenchymal transition, which leads to BRAF inhibitor resistance (Su et al., 2019).

BRAF/MEK inhibitor resistance in melanoma is associated with increased expression of EGFR (Ahn et al., 2017; Dratkiewicz et al., 2019). Resistance to BRAF inhibitors (dabrafenib or vemurafenib) results from *BRAF* amplification, *AKT* mutation, *N-RAS* mutation, *MEK1/MEK2* mutation, and high level of insulin like growth factor-1 receptor (IGF-1R) in *BRAF*^*V600E*^ mutant melanomas (Rizos et al., 2014, Figure 1E). *AKT1(Q79K)* mutation also confers resistance to BRAF inhibitors (vemurafenib or dabrafenib) via amplification of PI3K-AKT signaling (Shi et al., 2014). Resistance to BRAF inhibitors (vemurafenib or dabrafenib) results from alterations in MAPK pathway, such as MAP2K2, and melanocyte inducing transcription factor (MITF) (Van Allen et al., 2014). Melanoma cells can adapt to the drugs through phenotypic switching (plasticity), which results in resistance to targeted therapies such as BRAF and MEK inhibitors (Richard et al., 2016; Hartman et al., 2020). MITF, a regulator of melanoma cell plasticity, shows heterogeneous expression in cancer cell subpopulations (Vachtenheim and Ondrusova, 2015). Low expression of MITF expression is associated with invasion while high MITF expression favors cellular proliferation (Vachtenheim and Ondrusova, 2015). MITF regulates invasion of melanoma cells through negative feedback loop with Notch signaling (Golan and Levy, 2019). Therapy-resistant melanoma show low expression of MITF (Ahmed and Haass, 2018). High MITF level is found in more than 20% of melanomas following MAPK inhibitor treatment (Van Allen et al., 2014; Smith et al., 2016). MAPK inhibition leads to increased expression of MITF, which counteracts the effect of the MAPK inhibitor (Smith et al., 2019). Reactivation of MAPK signaling leads to activation of the PI3K-mTOR signaling pathway, which confers resistance to the BRAF inhibitors vemurafenib and dabrafenib (Welsh et al., 2016). Resistance to the BRAF inhibitor SB-590885 results from activation of IGF-1R/PI3K signaling (Villanueva et al., 2010). Resistance to PLX4720, an inhibitor of *BRAF*, results from upregulation of HDACs based on the fact that pan-HDAC inhibitors overcome resistance to PLX4720 (Lai et al., 2012). Trametinib-resistant melanoma cells show increased expression of HDACs 2/5/6/10/11 (Booth et al., 2017). A combination of vemurafenib and the MEK inhibitor trametinib increases the expression of HDAC8 in melanoma cells (Emmons et al., 2019). This increased expression of HDAC8 leads to the activation of MAPK signaling via receptor tyrosine kinases, such as EGFR and proto-oncogene MET, which confers resistance to the combination of BRAF inhibitor and MEK inhibitor (Emmons et al., 2019). It is therefore probable that HDAC8 is responsible for acquired resistance to the BRAF and MEK inhibitors. Figure 1E shows the mechanisms associated with resistance to BRAF inhibitors.

These reports suggest that targeting signaling pathways and/or HDACs may overcome resistance to BRAF inhibitors. Cancers are generally heterogeneous and multiclonal. An individual cancer reflects differences in mutations of various genes. Therefore, a combination of anti-cancer drugs is employed as anti-cancer therapy. Aberrant activation of the MAPK pathway is a major feature in most cases of melanoma (Dikshit et al., 2018). A combination of BRAF and MEK inhibitors has been employed for the treatment of metastatic melanomas harboring the *BRAF*^*V600E*^ mutation. The anti-tumor effects of these BRAF inhibitors are enhanced by co-administration of MEK inhibitors (Dummer et al., 2018). The combination of dabrafenib and trametinib results in stronger inhibition of activity of specific tyrosine kinases than does treatment with dabrafenib alone (Krayem et al., 2020). The combination of a BRAF inhibitor (dabrafenib) and a MEK inhibitor (trametinib) increases the expression of *KIT*, a tumor suppressor, and also induces alterations in *CCND1*, *RB1*, and *MET* in patients with *BRAF*^*V600E*^ metastatic melanoma (Louveau et al., 2019). The combination of cobimetinib (MEK inhibitor) and vemurafenib (BRAF inhibitor) improved progression-free survival compared to vemurafenib monotherapy in patients with *BRAF*^*V600*^ mutant metastatic melanoma in a phase 3 clinical trial (12.3 months vs. 7.2 months; Ascierto et al., 2016). In a phase III clinical of patients with advanced melanoma harboring the *BRAF*^*V600E*^ mutation, the combination of BRAF and MEK inhibitors (dabrafenib plus trametinib) increased the 3-year relapse-free survival rate compared to placebo treatment (58% vs. 39%) (Long et al., 2017).

Blockade of MAPK signaling pathway with BRAF and MEK inhibitors induces favorable responses, but most patients eventually develop resistance to these inhibitors. Melanoma patients harboring the *BRAF*^*V600E*^ mutation display primary resistance. Prolonged treatment with BRAF/MEK inhibitors induces acquired resistance (Atzori et al., 2020). These reports suggest that targeting molecular reprogramming induced by BRAF/MEK inhibitors is necessary to treat melanomas.

## The Roles of Hdacs in Melanoma Growth and Anti-Cancer Drug Resistance

**FIGURE 2:**
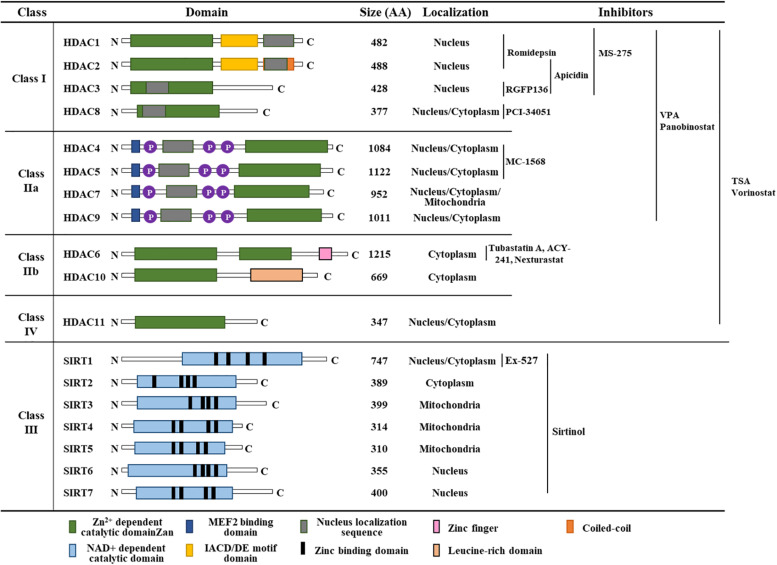
Classification of HDACs, functional domains, and HDAC inhibitors. TSA denotes trichostatin A. AA denotes amino acids.

HDACs deacetylate the lysine residues of histones that prevent transcription factor access (Guan et al., 2020). The HDAC family can be subdivided into four categories: Class I HDACs comprise HDAC 1, HDAC 2, HDAC 3, and HDAC 8, which are expressed in most tissues and localized in the nucleus. Class IIa HDACs (HDAC 4, HDAC 5, HDAC 7 and HDAC 9) are present in the nucleus and cytoplasm. Class IIb HDACs (HDAC 6 and HDAC 10) are expressed in a tissue-specific manner and localized in the cytoplasm. HDAC 11, the class IV HDAC, is present in the nucleus (Sahakian et al., 2015). Classes I, II, and IV HDACs require Zn^2+^ in their catalytic site, whereas class III HDACs require NAD^+^ for their deacetylase activity (Figure 2). Class III HDACs comprises seven sirtuin proteins (SIR1-7) and are homologous with the yeast protein SIR2. Inhibitors targeting classes I, II, and IV HDACs bind to the catalytic core of the Zn^2+^-binding site. Figure 2 shows classification, functional domains, and inhibitors of HDACs.

Chromatin state changes regulated by HDACs are closely associated with melanoma progression (Al Emran et al., 2018; Emran et al., 2019; Luo et al., 2020). Resistance to BRAF inhibitor results from increased expression of HDACs (Booth et al., 2017; Emmons et al., 2019). Downregulation of peroxisome proliferator-activated receptor γ coactivator 1-α (PGC1α) expression by H3K27me3 suppresses melanoma cell invasion (Luo et al., 2020). Aberrant expression, dysregulation of HDACs or imbalances between HDACs and histone acetyltransferases (HATs) promotes cancer progression (Krumm et al., 2016). Induced drug-tolerant melanoma cells show increased level of H3K9me3 and loss of H3K4me3/H3K27me3 (Al Emran et al., 2018). The loss of H3K4me3 in combination with increased DNA methylation of tumor suppressor genes leads to acquired anti-cancer drug resistance (Al Emran et al., 2018). The increased levels of H3K18ac and H3K27ac are responsible for multidrug resistance in renal cell carcinoma cells (Zhu et al., 2019). It is reasonable to conclude that an epigenetic regulator, such as HDACs/HATs, can regulate cancer cell growth and the responses to anti-cancer drugs.

HDACs regulate the expression levels of genes involved in melanoma cell proliferation (Kim et al., 2015; Chen et al., 2019). Malignant melanoma cells display high levels of HDAC1/2/3 compared to normal cells (Krumm et al., 2016). High expression of HDAC1 is seen in prostate cancers and breast cancers (Gameiro et al., 2016; Tang et al., 2017). Apicidin, an inhibitor of HDAC2 and HDAC3, decreases the expression of Notch1 by decreasing the level of H3Kac27 (Ferrante et al., 2020). Notch 1 signaling suppresses anti-tumor immunity by increasing the expression of TGF-β1 (Yang et al., 2018). Increased expression of HDAC2 is seen in human melanoma cells (Malme3M^R^) that have been made resistant to various anti-cancer drugs by repeated exposure to the anti-cancer drug celastrol (Kim et al., 2010). Downregulation of HDAC4 leads to apoptosis of head and neck cancer cells (Lee et al., 2018). HDAC5 promotes invasion of hepatocellular carcinoma cells by increasing the expression of hypoxia–inducible factor-1 (Ye et al., 2017). HDAC5 enhances the metastatic potential of neuroblastoma cells by decreasing the expression of CD9 via hypermethylation (Fabian et al., 2016). The hypermethylation of miR-589 promotes mesenchymal transition by upregulation of HDAC5 in non-small cell lung cancer cells (Liu et al., 2017). HDAC6, which is highly expressed in various melanoma cells, is necessary for invasion and metastasis of melanoma cells (Liu et al., 2016). HDAC6 deacetylates Lys-72 of extracellular signal-regulated kinase 1 (ERK1) and promotes ERK1 activity (Wu et al., 2018). HDAC6 binds to Tyrosine-protein phosphatase non-receptor type 1 (PTPN1), activates extracellular signal-regulated kinase 1/2 (ERK1/2), inhibits apoptosis, and promotes melanoma cell proliferation (Liu et al., 2018). HDAC7 regulates the level of acetyl-H3K27 and is necessary for maintaining cancer stem cells (Caslini et al., 2019). HDAC9 is highly expressed in most gastric cancer cells and plays on oncogenic role (Xiong et al., 2019). HDAC10 promotes angiogenesis by activating ERK1/2 phosphorylation (Duan et al., 2017). The class I and II HDAC inhibitor trichostatin A (TSA) decreases the expression of the genes involved in driving the extracellular signal-regulated kinase (ERK)1/2 oncogenic pathway (Mazzio and Soliman, 2018).

Valproic acid (VPA), an inhibitor of HDACs, binds to HDAC2 and enhances sensitivity to anti-cancer drugs (Kalal et al., 2019). HDAC2 binds to the cancer/testis antigens, such as CAGE, and leads to multi-drug resistance by decreasing p53 expression in melanoma cells (Kim et al., 2010, Figure 3A). HDAC5 confers resistance to tamoxifen by inducing deacetylation and nuclear localization of SOX9 (Xue et al., 2019). HDAC6 binds to tubulin β3 and confers resistance to anti-cancer drugs in Malme3M^R^ cells (Kim et al., 2015). Malme3M^R^ cells show low expression level of HDAC3 compared to parental anti-cancer drug sensitive melanoma cells (Malme3M) (Kim et al., 2014). Overexpression of HDAC3 enhances sensitivity to anti-cancer drugs by disrupting the interaction between HDAC6 and tubulin β3 (Kim et al., 2015, Figure 3B). HDAC3 decreases the expression of tubulin β3 by binding to its promoter sequences (Kim et al., 2015). HDAC3 suppresses the angiogenic potential of Malme3M^R^ cells by decreasing the expression levels of plasminogen activator inhibitor-1 (PAI-1) and vascular endothelial growth factor (VEGF) (Park et al., 2014, Figure 3B). HDAC3 forms a negative feedback loop with miR-326 and enhances sensitivity to anti-cancer drugs *in vitro* and *in vivo* (Kim et al., 2014). Thus, increasing HDAC3 expression may overcome resistance to anti-cancer drugs, including BRAF and MEK inhibitors. CAGE-derived ^269^GTGKT^273^ peptide binds to CAGE and enhances sensitivity to anti-cancer drugs in Malme3M^R^ cells (Kim et al., 2017). CAGE interacts with EGFR and human epidermal growth factor receptor 2 (HER2) to confer resistance to gefitinib and trastuzumab in Malme3M^R^ cells (Kim et al., 2016, Figure 3C). Thus, HDAC2-binding of CAGE can regulate the response to BRAF/MEK inhibitors. Table 1 shows the roles of HDACs in cancer cell proliferation, angiogenic potential, and metastasis.

**FIGURE 3:**
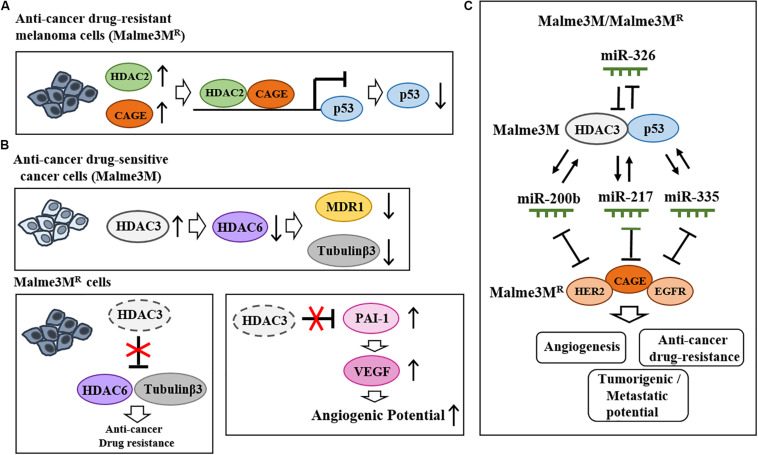
Effects of HDACs on the responses to anti-cancer drugs and melanoma growth. **(A)** HDAC2 binds to cancer/testis antigen CAGE and directly regulates the expression of p53 to confer resistance to various anti-cancer drugs in melanoma cells (upper). **(B)** In Malme3M Cells, HDAC3 decreases the expression levels of HDAC6, MDR1, and tubulin β3 (upper). In Malme3M^R^ cells, HDAC6 interacts with tubulin β3 and confers resistance to anti-cancer drugs (lower). HDAC3 negatively regulates angiogenic potential by decreasing the expression levels of PAI-1 and VEGF (lower). **(C)** HDAC3 forms a negative feedback loop with miR-326 and regulates the response to anti-cancer drugs as well as the tumorigenic and metastatic potential of melanoma cells. HDAC3 forms positive feedback loops with miR-200b, miR-217, and miR-335 in Malme3M cells. These miRNAs negatively regulate the expression of CAGE. CAGE interacts with EGFR and HER2 and confers resistance to anti-cancer drugs.

**TABLE 1:** Summary of functions of HDACs.

HDAC	Target molecule	Function	Cancer type	References
HDAC2	P53 ↓	Anti-cancer drug resistance ↑	Melanoma	Kim et al., 2010
HDAC3	Tubulin β3↓, HDAC6 ↓	Sensitivity to anti-cancer drugs ↑	Melanoma	Kim et al., 2015
HDAC3	PAI-1↓, VEGF ↓	Angiogenic potential ↓	Melanoma	Park et al., 2014
HDAC3	miR-326 ↓	Sensitivity to anti-cancer drugs ↑	Melanoma	Kim et al., 2014
HDAC5	HIF-1 ↑	Invasion potential ↑	Hepatocellular carcinoma	Ye et al., 2017
HDAC5	CD9 ↓	Metastatic potential ↑	Neuroblastoma	Fabian et al., 2016
HDAC5	–	Mesenchymal transition ↑	Non-small cell lung cancer	Liu et al., 2017
HDAC5	Deacetylation of SOX9	Anti-cancer drug resistance ↑	Breast cancer	Xue et al., 2019
HDAC6	Binds to ERK1/2	ERK1/2 activity ↑	HEK 293	Wu et al., 2018
HDAC6	ERK1/2 ↑	Apoptosis ↓, Proliferation ↑	Melanoma	Liu et al., 2018
HDAC6	Tubulin β3 ↑	Anti-cancer drug resistance ↑	Melanoma	Kim et al., 2015
HDAC7	Acetyl-H3K27 ↓	Cancer stem cell phenotypes ↑	Breast cancer	Caslini et al., 2019
HDAC8	c-Jun ↑	Anti-cancer drug resistance ↑	Melanoma	Emmons et al., 2019
HDAC9	–	Tumorigenic potential ↑	Gastric cancer	Xiong et al., 2019
HDAC10	PTPN22 ↓	ERK1/2 activity ↑, Angiogenic potential ↑	Endothelial cells	Duan et al., 2017

The HDAC inhibitors vorinostat and valproic acid (VPA) decrease the migration potential of *BRAF*^*V600E*^ mutant melanoma cells by increasing the expression of plasma membrane Ca^2+^ ATPase 4b (PMCA4b) (Hegedus et al., 2017). VPA increases acetylation of lysine residues of histone H3 at 9, 18, 23, and 27 at the promoter region of tissue type plasminogen activator (Larsson et al., 2012). Vorinostat induces H3K9 acetylation to exert anti-cancer effects in urothelial carcinoma cells (Eto et al., 2019), and decreases the tumorigenic potential of drug-resistant melanoma cells (Wang et al., 2018). The HDAC inhibitor panobinostat decreases PI3 kinase activity and increases the expression levels of apoptotic proteins such as BIM and NADPH oxidase activator (NOXA) (Gallagher et al., 2018). Panobinostat increases the acetylation of STAT3 at lysine 685 (Gupta et al., 2012). MS-275, an inhibitor of class I HDACs, increases H3K27ac and HDAC7 expression in breast cancer cells (Caslini et al., 2019). The class IIa-specific inhibitor MC-1568 increases the expression of Rb protein and the level of H3K27 at the Rb promoter (Rajan et al., 2018). The HDAC6-specific inhibitor ACY241 decreases the number of Treg cells (CD4^+^CD25^+^FoxP3^+^), but increases the number of activated CD8^+^ T cells by activating AKT signaling to induce anti-cancer effects against multiple myeloma (Bae et al., 2018). HDAC6-specific inhibitors (Tubastatin A and Nexturastat) suppress melanoma cell proliferation by increasing the expression levels of tumor-associated antigens (TAAs) and human leukocyte antigen (HLA) class I (Woan et al., 2015). High levels of TAAs activate CD8^+^ T cells to suppress cancer progression (Qu et al., 2018). Tubastatin A increases acetylation of Cystathionine γ-lyase (CSEγ) at lysine 73 (Chi et al., 2019). These reports suggest that HDAC inhibitors can regulate responses to anti-cancer drugs.

Class I HDAC inhibitors, such as VPA or MS-275, enhance the sensitivity of melanoma cells to the alkylating agents temozolomide, dacarbazine, and fotemustine by suppressing the double strand break (DSB) repair pathway by decreasing the expression levels of RAD51 and fanconi anemia complementation group D2 (FANCD2) (Krumm et al., 2016). The combination of trichostatin A (TSA) with etoposide increases the expression of p53 and reverses resistance to chemotherapy in melanoma cells (Monte et al., 2006). These reports imply a role for HDAC inhibitors in the response to BRAF/MEK inhibitors.

The combination of a BRAF inhibitor, encorafenib, and an HDAC inhibitor, panobinostat, synergistically induces caspase-dependent apoptotic cell death by inhibiting PI3 kinase activity and decreasing the expression levels of anti-apoptotic proteins (Gallagher et al., 2018). Vorinostat enhances sensitivity to dabrafenib and trametinib by increasing the level of reactive oxygen species (ROS) in anti-cancer drug-resistant melanoma cells (Wang et al., 2018). Vorinostat enhances the efficacy of BRAF/MEK inhibitors in *N-RAS* and *NF-1* mutant melanomas by suppressing DNA repair pathways (Maertens et al., 2019). The HDAC8 inhibitor PCI-34051 enhances sensitivity to BRAF inhibitors by increasing the acetylation of c-jun at lysine 273 (Emmons et al., 2019). GPCR-mediated yes associated protein (YAP) activation and receptor tyrosine kinase (RTK)-driven AKT signaling confer resistance to MEK inhibition. The HDAC inhibitor panobinostat prevents MEK inhibition from activating YAP and AKT signaling (Faiao-Flores et al., 2019). These reports indicate that a combination of an HDAC inhibitor and a BRAF/MEK inhibitor may offer clinical benefits in patients with metastatic melanoma.

## The Role of Immune Checkpoint in Melanoma Growth and Anti-Cancer Drug Resistance

Cancer cells evade immune surveillance and progress by activating immune checkpoint pathways that suppress the antitumor immune responses by CTLs. Vemurafenib-resistant (Vem^R^) cells display cross-resistance to melanoma antigen MART-specific CTLs and NK cells (Jazirehi et al., 2014). This indicates that lack of immune surveillance is responsible for resistance to BRAF inhibitors. Understanding the mechanisms of immune evasion is necessary for overcoming resistance to targeted and immune therapy. Immune checkpoint molecules, such as PD-1 and PD-L1, promote cancer progression by activating MDSCs and pro-tumorigenic tumor-associated macrophages (TAMs or M2 macrophages), while inhibiting CTLs and NK cells. High PD-L1 expression is common in malignant melanomas (Wang et al., 2019). The expression levels of PD-L1 and PD1 can predict the outcome of anti-PD1 immune therapy in malignant melanoma (Ugurel et al., 2020). The *BRAF*^*V600E*^ mutation leads to high PD-L1 level in a MEK-dependent manner (Feng D. et al., 2019).

**FIGURE 4:**
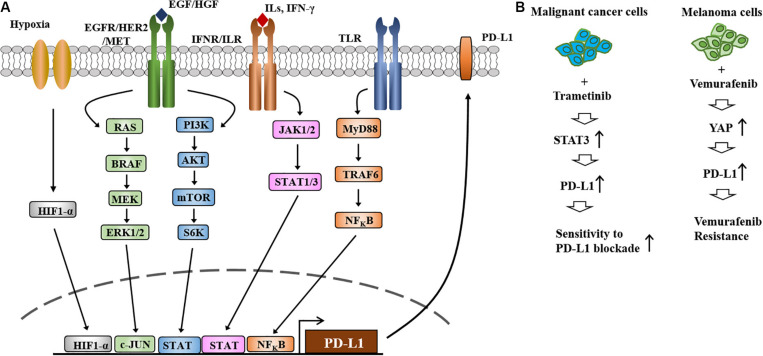
The expression and regulation of PD-L1and the role of PD-L1 in anti-cancer drug resistance. **(A)** Regulation of PD-L1 expression occurs at different levels. HIF-1α directly increases the expression of PD-L1 by binding to the promoter sequences of PD-L1. Toll-like receptor signaling increases the expression of PD-L1 by NF-kB. PI3K/AKT/mTOR and RAS/RAF/MEK/ERK signaling increase the expression of PD-L1 by activating C-Jun and STAT3. JAK/STAT signaling activated by IFN-γ increases the expression of PD-L1. **(B)** Treatment of metastatic melanomas with BRAF inhibitors or a combination of BRAF/MEK inhibitors leads to immune evasion (left). Increased expression of PD-L1 increases resistance to MEK inhibitors and EGFR-TKIs (left). MEKi denotes MEK inhibitor. EGFR-TKIs denote EGFR-tyrosine kinase inhibitors. Repeated exposure to vemurafenib increases the expression level of PD-L1, which in turn confers resistance to vemurafenib (right).

Activation of EGFR-STAT3 signaling is responsible for the increased expression of PD-L1 in melanoma cells (Ehexige et al., 2020; Li et al., 2020, Figure 4A). The MAPK and PI3K/AKT signaling pathways regulate the expression of PD-L1 (Li et al., 2019, Figure 4A). Melanoma extracellular vesicles increase the expression of PD-L1 via TLR4 signaling and suppress CTL activity (Fleming et al., 2019). Various transcription factors, such as HIF-1α, STAT3, C-JUN, ad NF-κB, regulate the expression of PD-L1 (Figure 4A).

Treatment with the MEK inhibitor trametinib increases the expression of PD-L1 via STAT3 activation, which in turn enhances sensitivity to PD-L1 blockade (Kang et al., 2019, Figure 4B). Resistance to the MEK inhibitor BAY86–9766 results from increased expression of EGFR and PD-L1 (Napolitano et al., 2019). Vemurafenib resistance results from the increased expression of PD-L1 by YAP, an effector of Hippo signaling, in melanoma cells (Kim et al., 2018, Figure 4B). These reports suggest that immune checkpoint molecules can determine melanoma growth and the response to anti-cancer drugs.

## HDAC Inhibitors Activate Immune Surveillance

**FIGURE 5:**
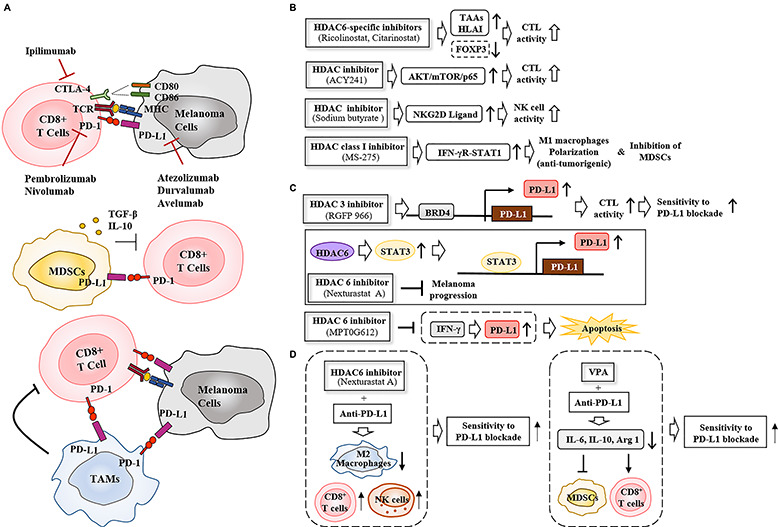
HDAC inhibitors enhance sensitivity to immune checkpoint inhibitors by regulating anti-tumor immune responses. **(A)** PD-1/PD-L1 interactions between cancer cells and CD8^+^ T cells suppress T cell activation, leading to tumor tolerance (upper). Ipilimumab, an anti-CTLA-4 antibody, disrupts the interaction between CTLA-4 and CD80/CD86, increasing production of pro-inflammatory cytokines and inducing T cell activation. MDSCs (middle) and TAMs (lower) suppress T cell activation via PD-1/PD-L1 interactions. MDSCs inhibit the function of CD8^+^ T cells by secreting TGF-β and IL-10. **(B)** HDAC inhibitors enhance CTL and NK cell activity, induce M1 macrophage polarization, and suppress the immune regulatory function of MDSCs. **(C)** HDACs regulate the PD-L1 expression to induce CTL activity or apoptosis. BRD4 denotes bromo domain protein 4. **(D)** HDAC inhibitors enhance sensitivity to PD-L1 blockade by activating CD8^+^T and NK cells while inactivating MDSCs and M2 macrophages. TAAs denote tumor associated antigens.

The tumor microenvironment consists of cancer cells and stromal cells (for example, cancer-associated fibroblasts, endothelial cells, and innate and adaptive immune cells). Cancer-associated fibroblasts induce phenotypic switching of melanoma cells into a mesenchymal-like phenotype and activate PI3K signaling to confer resistance to BRAF inhibitors (Seip et al., 2016). Therefore, cellular interactions within the tumor microenvironment may regulate the response to anti-cancer drugs. PD-1/PD-L1 interactions lead to immune evasion (tumor tolerance) by inactivating CD8^+^ T cells (Figure 5A). MDSCs interact with CD8^+^ T cells via PD-L1 and inactivate CD8^+^ T cells by secreting TGF-β and IL-10 (Fleming et al., 2018, Figure 5A). TAMs, which are activated by IFN-γ released by CD4^+^ T helper cells, inactivate CD8^+^T cells (Li et al., 2020, Figure 5A). Melanoma cells activate MDSCs, but inactivate CD8^+^ T cells via PD-L1 (Figure 5A). Specific depletion of pro-tumorigenic CD163^+^ M2 macrophages (TAMs) leads to infiltration of CTLs and tumor regression (Etzerodt et al., 2019).

The combination of DNA methyltransferase (DNMT) and histone deacetylase inhibitors decreases the number of MDSCs through type I IFN signaling and activates CD8^+^ T and NK cell signaling (Stone et al., 2017). This implies that epigenetic modifications regulate interactions between cancer cells and immune cells. HDAC6-selective inhibitors (ricolinostat and citarinostat) enhance the anti-tumor effects of CTLs in melanoma patients by decreasing the expression of Forkhead Box P3 (FOXP3) to suppress the functions of regulatory T cells (Laino et al., 2019, Figure 5B). The HDAC6 inhibitor ACY241 enhances the anti-tumor effects of antigen-specific CD8^+^ T cells by activating the AKT/mTOR/p65 pathways in solid tumors (Bae et al., 2018, Figure 5B). A combination of the HDAC inhibitor sodium butyrate and vemurafenib increases the expression of NK cell receptor (NKG2D)-ligand to enhance recognition of vemurafenib-treated melanoma cells by NK cells (Lopez-Cobo et al., 2018). MS-275 induces anti-tumorigenic M1 macrophage polarization through the IFN-γ receptor/STAT1 signaling pathway, and inhibits the function of MDSCs and eliminates antigen-negative cancer cells in a caspase-dependent manner (Nguyen et al., 2018, Figure 5B). The HDAC inhibitor vorinostat increases the expression levels of HLA classes I and II molecules on the cell surface to activate CTLs (Sun et al., 2019). These reports suggest that HDAC inhibitors may activate immune surveillance mechanism to suppress melanoma growth and enhance sensitivity to immune checkpoint inhibitors.

## HDAC Inhibitors Enhance the Efficacy of Immune Checkpoint Inhibitors

Immune checkpoint inhibitors, such as anti-cytotoxic T Lymphocyte associated protein- 4 (CTLA-4) antibody (Ipilimumab) and anti-PD-L1 antibodies (atezolizumab, druvalumab, and avelumab) have shown some clinical benefits in the treatment of patients with advanced-stage metastatic melanoma. The overall response to atezolizumab was 30% among 43 melanoma patients in a phase I clinical trial (Hamid et al., 2019). Anti-PD1 antibodies, such as nivolumab and pembrolizumab, are also widely used to treat advanced melanoma (Fujimura et al., 2019).

Epigenetic modifications regulate expression of the genes involved in immune surveillance. Bromodomain and extra-terminal region (BET) protein recognizes acetylated lysines of histones and non-histone proteins (Rajendran et al., 2019). BET inhibitors suppress melanoma growth by decreasing the expression of PD-L1 while activating CD8^+^ T cells (Erkes et al., 2019). HDAC6 increases the expression of PD-L1 through STAT3 signaling, and selective inhibition of HDAC6 suppresses cancer progression *in vivo* (Lienlaf et al., 2016, Figure 5C). The inhibition of HDAC6 by MPT0G612 prevents IFN-γ from increasing the expression of PD-L1 and induces apoptosis by suppressing autophagy (Chen et al., 2019, Figure 5C). RGFP966 increases the expression of PD-L1 in dendritic cells, and the combination of RGFP966 with anti-PD-L1 antibody suppresses murine lymphoma growth (Deng et al., 2019, Figure 5C).

The effect of immune checkpoint blockade is compromised by activation of MDSCs. A combination of the HDAC inhibitor VPA and anti-PD-L1 antibody inhibits functioning of MDSCs by decreasing the expression levels of IL-10, IL-6, and Arginase I (ARG1) while activating CD8^+^ T cells (Adeshakin et al., 2020, Figure 5D). The HDAC6 inhibitor nexturastat A improves the efficacy of anti-PD-1 antibody by decreasing the number of pro-tumorigenic M2 macrophages (TAMs) while increasing the number of tumor infiltrating NK cells and CD8^+^ T cells (Knox et al., 2019, Figure 5D). PD-1 blockade increases the expression of PD-L1 via pro-inflammatory cytokines such as IFN-γ (Knox et al., 2019). Nexturastat A prevents anti-PD-1 antibody from increasing the expression of PD-L1 (Knox et al., 2019). These reports indicate that HDAC inhibitors enhance the responses to immune checkpoint inhibitors by activating immune surveillance.

## Conclusion and Perspectives

To better understand the mechanisms of resistance to BRAF/MEK inhibitors in melanoma, identification of molecular signatures associated with resistance is necessary. Establishment of melanoma cell lines that are resistant to these inhibitors will make it possible to identify molecular signatures that may serve as targets for the development of anti-melanoma therapies.

MicroRNAs (miRNAs) are small non-coding RNAs that play important roles in cellular proliferation, anti-cancer drug resistance and cancer progression (Kim et al., 2017). miR-22 directly binds to the 3′ UTR of HDAC6 and suppresses cervical cancer cell proliferation (Wongjampa et al., 2018). miRNAs that target specific HDACs can overcome resistance to targeted and immune therapy. Downregulation of miR-589 promotes cancer malignancy by increasing PD-L1 expression level (Liu et al., 2017). miR-146a, which is increased in metastatic melanoma, induces immune evasion of melanoma cells (Mastroianni et al., 2019). The combination of a miR-146a inhibitor and anti-PD-L1 improves survival in a mouse model of melanoma (Mastroianni et al., 2019). It is necessary to identify miRNAs that bind to the 3′ UTR of PD-L1 and/or PD-1. These miRNAs can be developed as anti-melanoma therapies in combination with HDAC inhibitors and immune checkpoint inhibitors.

Epigenetic modifications regulate cancer progression and anti-cancer drug resistance. Epigenetic modifications are reversible and dynamic. Thus, targeting HDACs has emerged as an attractive strategy for the treatment of various cancers. Reportedly, HDACs regulate the expression levels of immune checkpoint molecules. Thus, targeting HDACs may prove to be an effective strategy to overcome resistance to immune checkpoint blockade.

The FDA has approved four HDAC inhibitors for use in cancer patients. These inhibitors are Vorinostat (hydroxamic acid family), Romidepsin (cyclic peptide family), Belinostat (hydroxamic acid family), and Panobinostat (hydroxamic acid family). These inhibitors have been approved for the treatment of cutaneous T-cell lymphoma and peripheral T cell lymphoma. In a phase II clinical trial, some patients with advanced melanoma displayed an early response to vorinostat. However, the disease state in most of these patients was stable (Haas et al., 2014). Vorinostat therapy has many side effects, including fatigue, nausea, and lymphopenia (Haas et al., 2014). Vorinostat and the proteasome inhibitor marizomib have synergistic effects when used together in cancer cell lines derived from melanoma patients and are well-tolerated by melanoma patients (Millward et al., 2012). The combination of belinostat (an inhibitor of HDACI and HDACII) with cisplatin and etoposide lea to hematologic toxicity in a phase I clinical trial of advanced small cell lung cancer patients (Balasubramaniam et al., 2018). The combination of romidepsin and the DNA methyl transferase I inhibitor 5-aza-deoxycydine displayed dose-limiting toxicity, including grade 4 thrombocytopenia, grade 4 neutropenia, and pleural effusion (O’Connor et al., 2019). The overall response rate to a combination of romidepsin and 5-aza-deoxycydine in T-cell lymphoma patients was 55% (O’Connor et al., 2019). A combination of romidepsin and the BET inhibitor IBET151 increases the expression of IL-6 and the number of antigen-specific CD8^+^ cells during vaccination for the treatment of melanoma (Badamchi-Zadeh et al., 2018). Panobinostat (a pan-deacetylase inhibitor) showed a very low response rate and a highly toxic effects in phase I trial of patients with metastatic melanoma (Ibrahim et al., 2016). Panobinostat treatment was associated with high rates of nausea, vomiting, and fatigue in phase 1 trial of metastatic melanoma patients (Ibrahim et al., 2016). The combination of panobinostat and the proteasome inhibitor carfilzomib had adverse effects, including thrombocytopenia (41%), fatigue (17%), and nausea/vomiting (12%) in a phase I trial of 32 patients with multiple myeloma (Kaufman et al., 2019). The objective response rate (ORR) and clinical benefit rate were 63 and 68%, respectively in that same phase I trial of 32 patients with multiple myeloma (Kaufman et al., 2019). Quisinostat, hydroxamate-based HDAC inhibitor, targets both class I and II HDACs. According to the results of a phase I clinical trial, quisinostat shows strong antitumor effect and is well-tolerated in metastatic melanoma patients (Venugopal et al., 2013). Table 2 describes clinical trials of HDAC inhibitors in various cancers, including melanoma.

**TABLE 2:** Clinical trials of HDAC inhibitors: characteristics of clinical trials registered in https://clinicaltrials.gov.

Title	Treatment	Characteristics	Condition	Phase	Dates	NCT Number
Vorinostat in Treating Patients With Metastatic or Unresectable Melanoma	Vorinostat	• Enrollment : 32 patients with advanced melanoma • Administration : dose of 400 mg for 28 consecutive days per cycle • Adverse events : fatigue, nausea, lymphopenia, and hyperglycemia • Outcome : 2 partial response and 16 stable disease (median PFS of 5 months), 14 progressive disease (median PFS of 4 months)	Melanoma	Phase 2	• Study Start : September 2005 • Study Completion : June 2013	NCT00121225
NPI-0052 and Vorinostat in Patients With Non-small Cell Lung Cancer, Pancreatic Cancer, Melanoma or Lymphoma	NPI-0052 (marizomib) Vorinostat	• Enrollment: 22 patients with melanoma, pancreatic carcinoma or Non-small Cell Lung Cancer (NSCLC) • Administration: doses of weekly marizomib in combination with vorinostat 300 mg daily for 16 days in 28 day cycles • Adverse events : fatigue, nausea, vomiting, diarrhea, anorexia, dyspnoea, headache, infusion site pain • Outcome : 61% stable disease, 39% partial response	Non-Small Cell Lung CancerPancreatic CancerMelanoma LymphomaMultiple Myeloma	Phase 1	• Study Start : March 2008 • Study Completion : January 2010	NCT00667082
A Phase I Study of Belinostat in Combination With Cisplatin and Etoposide in Adults With Small Cell Lung Carcinoma and Other Advanced Cancers	Belinostat Cisplatin Etoposide	• Enrollment : 28 patients with histologically or cytologically confirmed cancers for which there is no known standard therapy capable of extending life expectancy • Administration : doses of belinostat 400 mg/m^2^ on days 1and 2, cisplatin 80 mg/m^2^ on day 2, and etoposide 100mg/m^2^ daily times 3 on days 2 to 4 • Outcome : 11 partial response, 13 stable disease, and 4 progressive disease	Carcinoma NeuroendocrineSmall Cell Lung CarcinomaMalignant Epithelial Neoplasms	Phase 1	• Study Start : July 1, 2009 • Study Completion : April 20, 2018	NCT00926640
Panobinostat (LBH589) in Patients With Metastatic Melanoma	Panobinostat	• Enrollment : 16 patients with metastatic melanoma that is amenable to serial biopsies • Administration : doses of LBH589 three days a week(Monday, Wednesday and Friday) every other weak • Adverse events : thrombocytopenia, lymphocytopenia, LFT elevation, hypophosphatemia, hypokalemia Outcome : 27% stable disease, 73% progressive disease	Malignant Melanoma	Phase 1	• Study Start : February 2010 • Study Completion : March 13, 2017	NCT01065467
A Safety and Dose-finding Study of JNJ-26481585 for Patients With Advanced Solid Malignancies and Lymphoma.	Quisinostat	• Enrollment : 22 with advanced solid tumors or lymphomas that were refractory to standard therapy • Administration : doses of quisinostat once a day for 21 days cycle • Adverse events: fatigue, cardiac disorder, decreased appetite, ventricular tachycardia, lethargy, and vomiting • Outcome: 3 partial response, and 6 stable disease	LymphomaNeoplasmsa	Phase 1	• Study Start : August 2007 • Study Completion : September 2011	NCT00677105
Selective HDAC6 Inhibitor ACY-241 in Combination With Ipilimumab and Nivolumab	ACY-241 Nivolumab Ipilimumab	• Enrollment : 1 patient with advanced melanoma • Administration : doses of ACY-241 in combination with ipilimumab and nivolumab every 3 weeks for 4 doses each during a 12-week	Malignant Melanoma	Phase 1	• Study Start : September 30, 2016 • Study Completion : April 7, 2017	NCT02935790
HDAC Inhibitor Vorinostat in Resistant BRAF V600 Mutated Advanced Melanoma	Vorinostat	• Enrollment : 21 patients with BRAF V600 mutated melanoma who developed resistance to BRAFi and/or BRAFi+MEKi • Administration : dose of vorinostat 360 mg once daily	Melanoma Skin Neoplasms	Phase 1Phase 2	• Study Start : June 2016 • Primary Completion : October 2019	NCT02836548

To date, there have been no successful clinical trials involving a combination of HDAC inhibitors and immune checkpoint inhibitors. The HDAC-selective inhibitors that are currently in use have off-target effects. To overcome these off target effects, it is necessary to design HDAC-specific inhibitor based on the structure of each HDAC. Identification of proteins that interact with individual HDACs may make it possible to devise new anti-melanoma therapies. We previously reported that CAGE-binding peptide prevents CAGE from binding to GSK3β and enhances sensitivity to anti-cancer drugs (Kim et al., 2017). Identification of proteins that interact with individual HDACs is necessary for development of anti-melanoma therapies. Peptides that bind to each HDAC and prevent interactions between each HDAC and its binding partner may circumvent off-target effects and enhance sensitivity to targeted and immune therapies.

Due to tumor heterogeneity and plasticity, combination therapy is required for the treatment of cancers, including melanomas. HDACs play major roles in the regulation of immune checkpoint molecules, cancer cell proliferation, and activation of oncogenic signaling pathways. It is reasonable to conclude that HDAC inhibitors in combination with targeted therapies and immune therapies can be employed as anti-melanoma therapies.

## Author Contributions

DJ wrote the manuscript. MY made the figures and tables. HJ helped in editing. YK and HJ provided intellectual output in the manuscript.

## Conflict of Interest

The authors declare that the research was conducted in the absence of any commercial or financial relationships that could be construed as a potential conflict of interest.
